# A Semi-Dominant Mutation in the General Splicing Factor SF3a66 Causes Anterior-Posterior Axis Reversal in One-Cell Stage *C. elegans* Embryos

**DOI:** 10.1371/journal.pone.0106484

**Published:** 2014-09-04

**Authors:** Mohammad R. Keikhaee, Eric B. Nash, Sean M. O'Rourke, Bruce Bowerman

**Affiliations:** Institute of Molecular Biology, University of Oregon, Eugene, Oregon, United States of America; University of Sheffield, United Kingdom

## Abstract

Establishment of anterior-posterior polarity in one-cell stage *Caenorhabditis elegans* embryos depends in part on astral microtubules. As the zygote enters mitosis, these microtubules promote the establishment of a posterior pole by binding to and protecting a cytoplasmic pool of the posterior polarity protein PAR-2 from phosphorylation by the cortically localized anterior polarity protein PKC-3. Prior to activation of the sperm aster, the oocyte Meiosis I and II spindles assemble and function, usually at the future anterior pole, but these meiotic spindle microtubules fail to establish posterior polarity through PAR-2. Here we show that a semi-dominant mutation in the general splicing factor SF3a66 can lead to a reversed axis of AP polarity that depends on PAR-2 and possibly on close proximity of oocyte meiotic spindles with the cell cortex. One possible explanation is that reduced levels of PKC-3, due to a general splicing defect, can result in axis reversal due to a failure to prevent oocyte meiotic spindle microtubules from interfering with AP axis formation.

## Introduction

Asymmetric cell division is a fundamental process that generates cell fate diversity [1], [2]. In the one-cell stage *C. elegans* embryo, anterior-posterior axis establishment is followed by an asymmetric cell division that produces posterior and anterior daughters that differ in size, fate and cell cycle timing [3]. Genetic investigation of this first mitotic division has led to the identification of conserved cell polarity regulators, but the mechanisms that establish AP polarity remain incompletely understood [4].

Before mitosis, the *C. elegans* zygote lacks polarity along its long axis [5]. The subsequent establishment of AP polarity may depend in part on astral microtubules that, after completion of Meiosis I and II, radiate out from the sperm-donated centrosomes [6], although this issue remains controversial and could depend on cytoplasmic microtubules not directly associated with centrosomes [7]. The sperm aster and associated sperm pronucleus are located at one pole, with Meiosis I and II usually producing polar bodies at the opposite pole. Prior to the completion of oocyte Meiosis I and II and the subsequent sperm aster activation, cortical polarity proteins including PAR-3 are evenly distributed throughout the cortex, as is the actomyosin cytoskeleton. Upon sperm aster activation, poorly understood centrosomal cue(s) trigger contraction of the actomyosin cytoskeleton away from the cortical site overlying the sperm aster [8]–[10], with passive advection moving cortical PAR-3 away from this site [11]. The initially cytoplasmic posterior polarity protein PAR-2 then localizes to the cortical region vacated by PAR-3, and subsequent mutually antagonistic interactions between PAR-2 and PAR-3 lead to the expansion of a posterior cortical PAR-2 domain and the eventual establishment of a fully polarized zygote [12].

Although the sperm-donated centrosome may provide additional cues, the astral microtubules it nucleates, or other microtubules, appear to play a critical role in specifying the posterior pole [6], [7]. They do so at least in part by binding to PAR-2 and protecting it from phosphorylation by PKC-3, the atypical protein kinase C that associates with PAR-3 as part of the anterior cortical PAR complex [13]. Phosphorylation of PAR-2 inhibits its cortical localization, while microtubule binding of PAR-2 protects it from PKC-3 and positions unphosphorylated PAR-2 near the region of the cortex vacated by PAR-3 upon activation of the sperm aster.

The oocyte Meiosis I and II spindles function prior to sperm aster activation and usually are positioned at the future anterior pole [14]. Thus these microtubules also could bind to and protect PAR-2 from PKC-3 phosphorylation. Indeed, in wild-type zygotes PAR-2 transiently accumulates at the cortex overlying the oocyte meiotic spindles [13], and mutational inactivation of the *C. elegans* anaphase promoting complex results in arrest of Meiosis I at metaphase and a partial reversal of AP polarity, with PAR-2 localized to the anterior and not the posterior pole [15].

How wild-type zygotes prevent oocyte meiotic spindle microtubules from interfering with AP axis formation is not well understood. Here we report our analysis of a general splicing factor mutant called *repo-1(or430*ts*)* in which progression through oocyte meiosis I and II is normal but AP polarity is nevertheless reversed in roughly half the mutant zygotes. Oocyte meiotic spindles promote, and PKC-3 opposes, this reversal. It is possible that one role for PKC-3 is to prevent oocyte meiotic spindles from interfering with AP axis formation, and that reduced expression of PKC-3 due to a general splicing defect can lead to axis reversal.

## Results

### Reversed AP polarity in *or430*ts mutants

**Figure 1:**
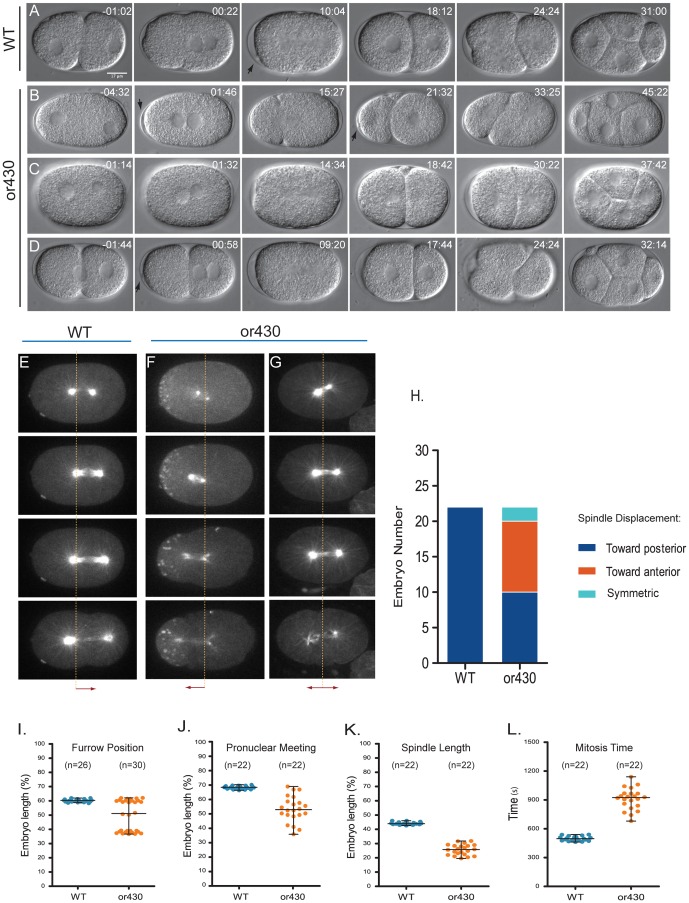
Reversed polarity of P0 asymmetric cell division in *or430*ts zygotes. Time-lapse DIC images of wild type (A) and *or430*ts mutants (B–D), showing *or430*ts embryos with reversed (B), symmetric (C) and normal (D) cell division. Arrows indicate polar bodies. (E–G) Time-lapse confocal images of P_0_ mitotic spindle orientation in wild-type and *or430*ts zygotes expressing a GFP fusion to ß-tubulin (and a GFP fusion to PIE-1 in F that labels P granules and cytoplasmic PIE-1)). Vertical lines in each image indicate 50% egg length. (H) P_0_ mitotic spindle orientations in wild-type and *or430*ts zygotes (each n = 22). (I) P_0_ cleavage furrow position along AP axis in wild-type (n = 26) and *or430*ts zygotes (n = 30). (J) Pronuclear meeting site along AP axis in wild-type (n = 22) and *or430*ts zygotes (n = 22). (K) Maximum P_0_ mitotic spindle length in wild-type and *or430*ts zygotes (n = 22). (L) Duration of P_0_ mitosis from pronuclear meeting to cleavage furrow ingression in wild-type and *or430*ts zygotes (each n = 22). In this and subsequent figures, t = 0 corresponds to pronuclear meeting.

In a screen for temperature-sensitive, embryonic-lethal *C. elegans* mutants [16], we isolated *or430*ts, which frequently exhibited a striking reversal in the position of the first mitotic spindle along the anterior-posterior axis, when live embryos were examined using time-lapse microscopy (Figure 1A, 1B, 1H, 1L). Normally the one-cell stage embryo, called P_0_, assembles a mitotic spindle that becomes displaced toward the posterior pole, and the posterior daughter, P_1_, is smaller than the anterior daughter, AB. Subsequently, AB enters the next round of mitosis before P_1_ (3).

By contrast, in just under half of the embryos examined, the first mitotic spindle in *or430*ts mutants was displaced toward the anterior pole, as marked by the oocyte polar bodies, resulting in smaller anterior and larger posterior two-cell stage blastomeres (Figure 1A, 1B, 1H, 1L). Moreover, the anteriorly positioned P_1_-like daughter entered mitosis later than the posteriorly positioned AB-like daughter, further suggesting a reversal in AP polarity (Figure 1B). In slightly less than half the mutants, the P_0_ mitotic spindle and cleavage furrow were displaced as in wild-type embryos toward the posterior pole (Figure 1D, 1G, 1H, 1L), defined as the pole opposite the polar body; in about 10% of the mutant embryos P_0_ produced equal sized daughters (Figures 1C, 1G, 1H, 1L) that divided synchronously (Figure 1C). Thus there appears to be a loss of AP polarity in a small fraction of *or430*ts embryos.

### Pleiotropic defects in *or430*ts mutants

**Figure 2:**
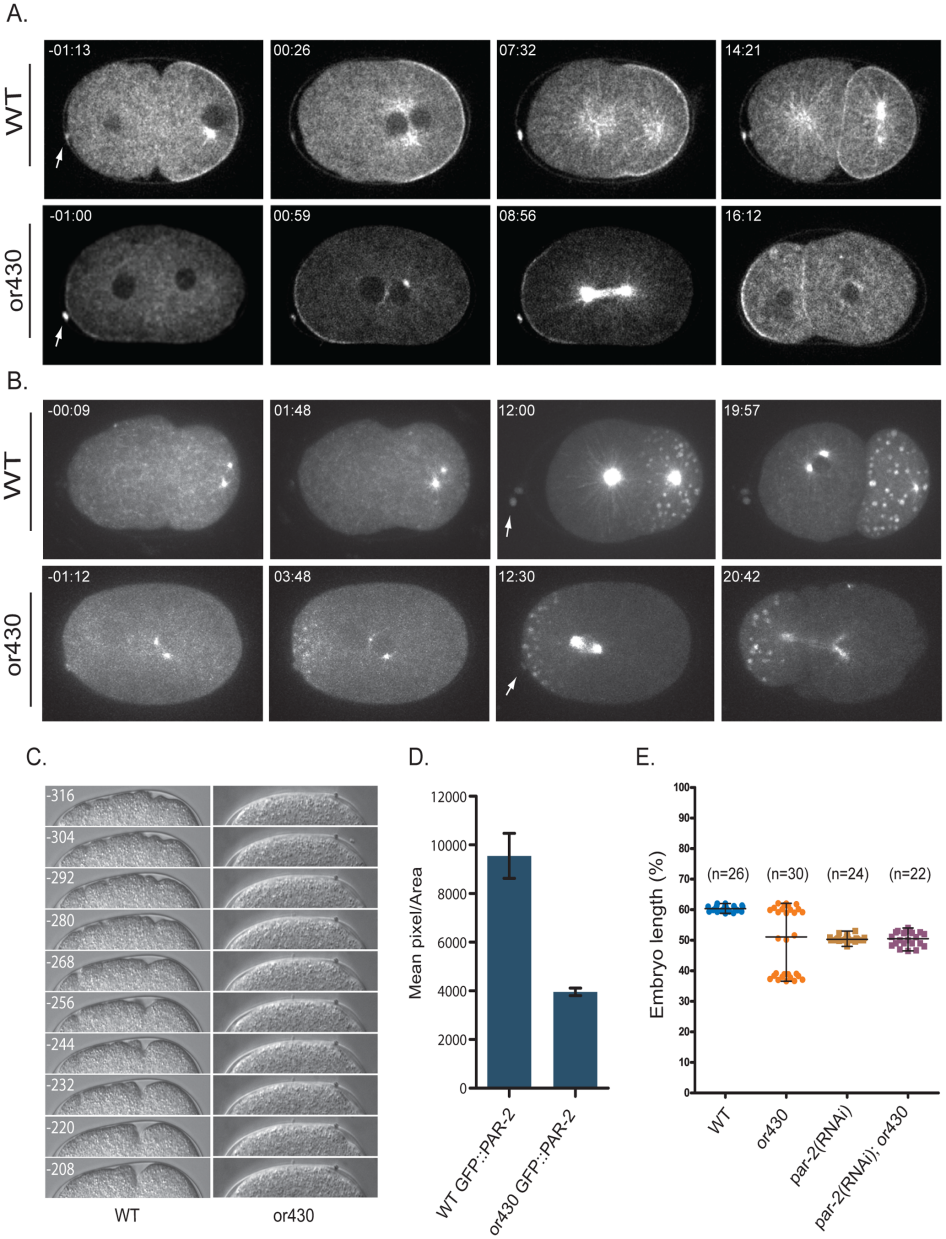
AP axis reversal in *repo-1(or430*ts*)* zygotes. (A) Time-lapse confocal images of wild type and *or430*ts zygotes expressing GFP fusions to ß-tubulin and PAR-2. (B) Time-lapse confocal images of wild type and *or430*ts zygotes expressing GFP fusions to ß-tubulin and PIE-1. (C) Kymographs of pseudocleavage furrow movement in wild-type zygotes and its absence in *or430*ts zygotes. Time in seconds before pronuclear meeting for both wild-type and *or430*ts are shown in wild-type images. (D) Quantification of GFP: PAR-2 levels in wild-type (n = 10) and *or430*ts (n = 10) zygotes; see Materials and Methods for details. (E) Location along AP axis of P0 cytokinesis furrow ingression in wild-type and mutant zygotes. Arrows indicate polar bodies. P<0.001 for an independent t test to compare the difference of the furrow position for *or430* versus *or430; par-2(RNAi)*. Data for WT and *or430*ts are same as in Figure 1I.

We then further compared the P_0_ cell divisions in *or430*ts and wild-type embryos. First, we measured the position along the embryos' long axis where the oocyte and sperm pronuclei met after migration. In wild-type, pronuclei congress near the posterior pole. By contrast, in many *or430*ts embryos pronuclei met near the midpoint of the long axis, with the position of their meeting being highly variable. In other mutants that fail to establish AP polarity, pronuclei also meet in the middle [17], further indicating that *or430*ts mutants have polarity defects. We also found that membrane invaginations were greatly reduced and pseudocleavage furrowing absent during the one-cell stage in most (13/20) *or430*ts mutant zygotes (Figure 2C). As the actomyosin cytoskeleton is required for these membrane dynamics and for the establishment of AP polarity [17], these defects in *or430*ts mutants also are consistent with the observed polarity defects. Finally, we also observed that the centrosomes and associated sperm pronucleus were often abnormally far from the cell cortex after the completion of meiosis I and II (Figure 1C; data not shown), that *or430*ts P_0_ mitotic spindles were abnormally small, failing to elongate normally (Figure 1K), and that embryos progressed more slowly through mitosis compared to wild-type (Figure 1L). As proximity to the cortex of the sperm pronucleus-associated centrosomes is important for proper establishment of AP polarity [17], these defects in centrosome position and mitotic spindle assembly might contribute to the observed polarity defects in *or430*ts mutants.

### *or430*ts AP polarity reversal requires PAR-2

We next used genetic crosses to introduce the *or430*ts mutation into strain backgrounds that express translational fusions of GFP to the posterior cortical PAR-2 protein, and to PIE-1, a component of the germline P granules that localize to the posterior cytoplasm of P0 before it divides (Figures 2A, 2B). In just under half of *or430*ts mutants, both PAR-2 and PIE-1 were reversed with respect to their normal localization relative to the occyte polar bodies: GFP::PAR-2 was present at the anterior instead of the posterior cortex, and GFP::PIE-1-labeled P granules were localized to the anterior instead of posterior cytoplasm. In a roughly equal number of *or430*ts mutants, PAR-2 and P granules were both posteriorly localized, as in wild-type. Finally, in about 10% of the mutant embryos PAR-2 was absent from the cortex and P granules were distributed throughout P_0_ (data not shown), again suggesting a loss of polarity in some embryos. To determine if the polarity reversals depend on PAR-2 mis-localization to the anterior pole, we used RNAi to reduce *par-2* function and found that P_0_ divided equally in all *par-2(RNAi); or430*ts double mutants, as in *par-2(-)* single mutants (Fig. 2B). We conclude the polarity reversals depend on PAR-2. Because the AP polarity is reversed in ∼40% of *or430*ts mutants, we named the locus *repo-1* for reversed polarity.

### *or430*ts is a semi-dominant allele of the general splicing factor SF3a66

We next tested whether the *or430*ts mutation is dominant or recessive, scoring embryonic viability after heterozygous *unc-30(e191) repo-1(or430ts)/+* hermaphrodites matured to adulthood at 26°C. 79% of these embryos hatched (n = 364) and 19% of the hatched larvae (n = 290) were Unc. We conclude that *or430*ts has a semi-dominant maternal-effect, with little if any recessive zygotic-effect embryonic lethality.

**Figure 3:**
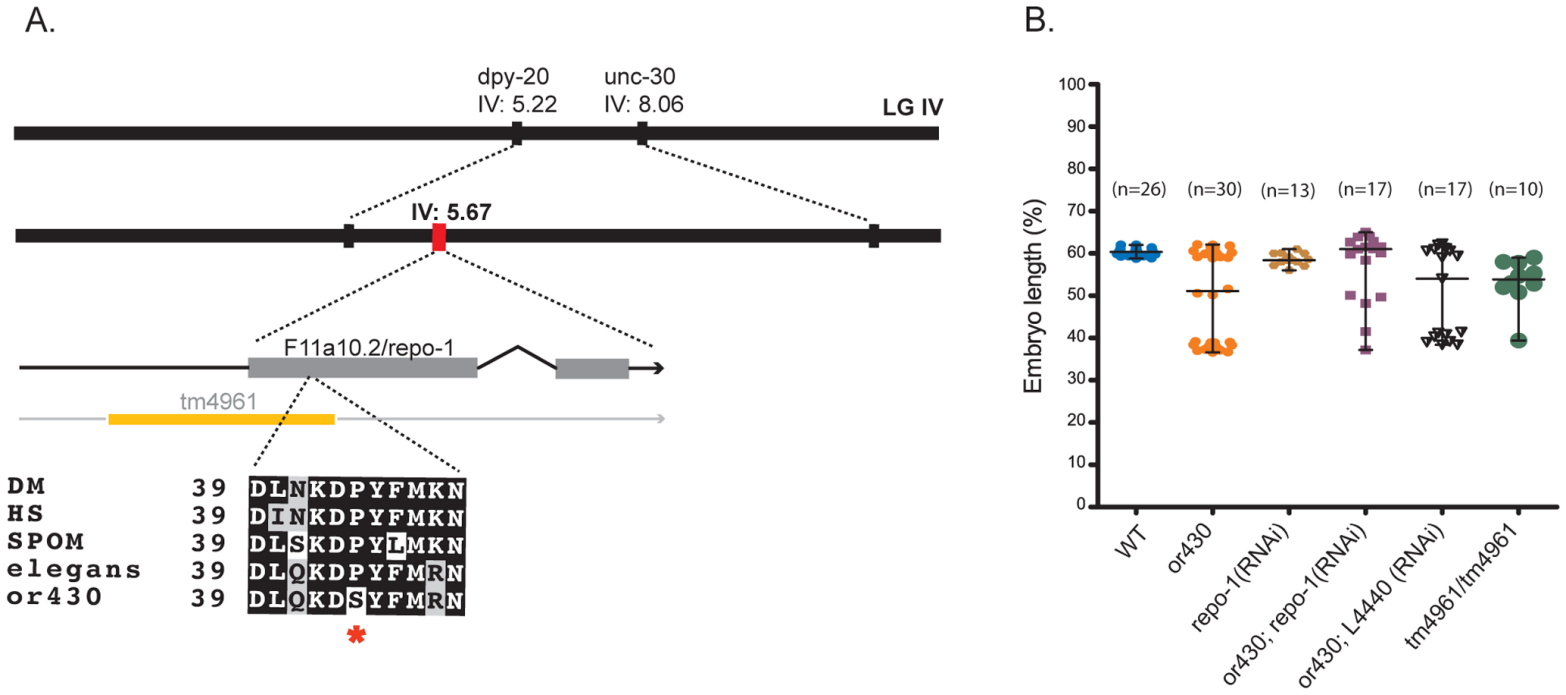
Identification of *repo-1(or430*ts*)* causal mutation in the SF3a66 *C. elegans* ortholog F11a10.2. (A) Schematic of *repo-1* map position on chromosome IV and partial amino acid sequences of predicted proteins in wild-type and *or430*ts F11a10.2/*repo-1*, and in fly, human and fission yeast orthologs. Location of *tm4961* deletion indicated; whole genome sequencing of *repo-1(or430*ts*)* revealed no sequence changes in the neighboring gene, *lex-1*, which also is disrupted by the *tm4961* deletion (data not shown). (B) Loss of polarity reversal after RNAi knockdown of F11a10.2 in *repo-1(or430*ts*)* mutants, and in *repo-1(tm4961)/repo-1(tm4961)* mutants, assessed by measuring P_0_ cleavage furrow position. Note that the single embryo with an apparent reversal in *repo-1(tm4961)* mutants may represent an example of a posteriorly positioned polar body, which occurs at low frequency in wild-type embryos. WT and *or430*ts data are same as in Figure 1I. L4440 refers to the empty vector used as a negative control for feeding RNAi.

To identify the causal mutation, we first used visible markers to map *or430*ts to about +5.7 map units on linkage group IV (Fig. 3). We then sequenced amplified DNA from genes in the region and found a mis-sense mutation in F11A10.2 at nucleotide position 130 (ccg to tcg), corresponding to amino acid 44 (Pro to Ser); this nucleotide change was not present in the parental strain used for mutagenesis (Fig. 3A). We next performed a complementation test with a 556 base pair lethal deletion allele, *tm4961*, that removes the 5′ sequences of the *repo-1/*F11A10.2 locus and 3′ sequences of an adjacent locus: *or430*ts failed to complement *tm4961* (Fig. 3A), confirming that *repo-1(or430*ts*)* is an allele of F11A10.2. Because genome-wide RNAi screens have reported that reducing *repo-1* function can result in embryonic-lethality with no early embryonic cell division defects, we used RNAi to knock down *repo-1* in homozygous *or430ts* mutants. We found that these embryos were still inviable, as expected, but no longer exhibited a polarity reversal (Fig. 3B), supporting our conclusion that the F11A10.2 mutation is responsible for the semi-dominant phenotype. Moreoever, we also did not observe polarity defects in embryos from homozygous *repo-1(tm4961)* mutants. We conclude that the allele *repo-1(or430*ts*)* is semi-dominant and neomorphic. The F11A10.2/*repo-1* gene encodes a 200 amino acid protein, the ortholog of human SF3a66 that is a component of U2 snRNPs involved in pre-mRNA splicing and can bundle microtubules independently of its splicing factor role [18].

### *repo-1(or430*ts*)* mutants progress normally through oocyte Meiosis I and II

**Figure 4:**
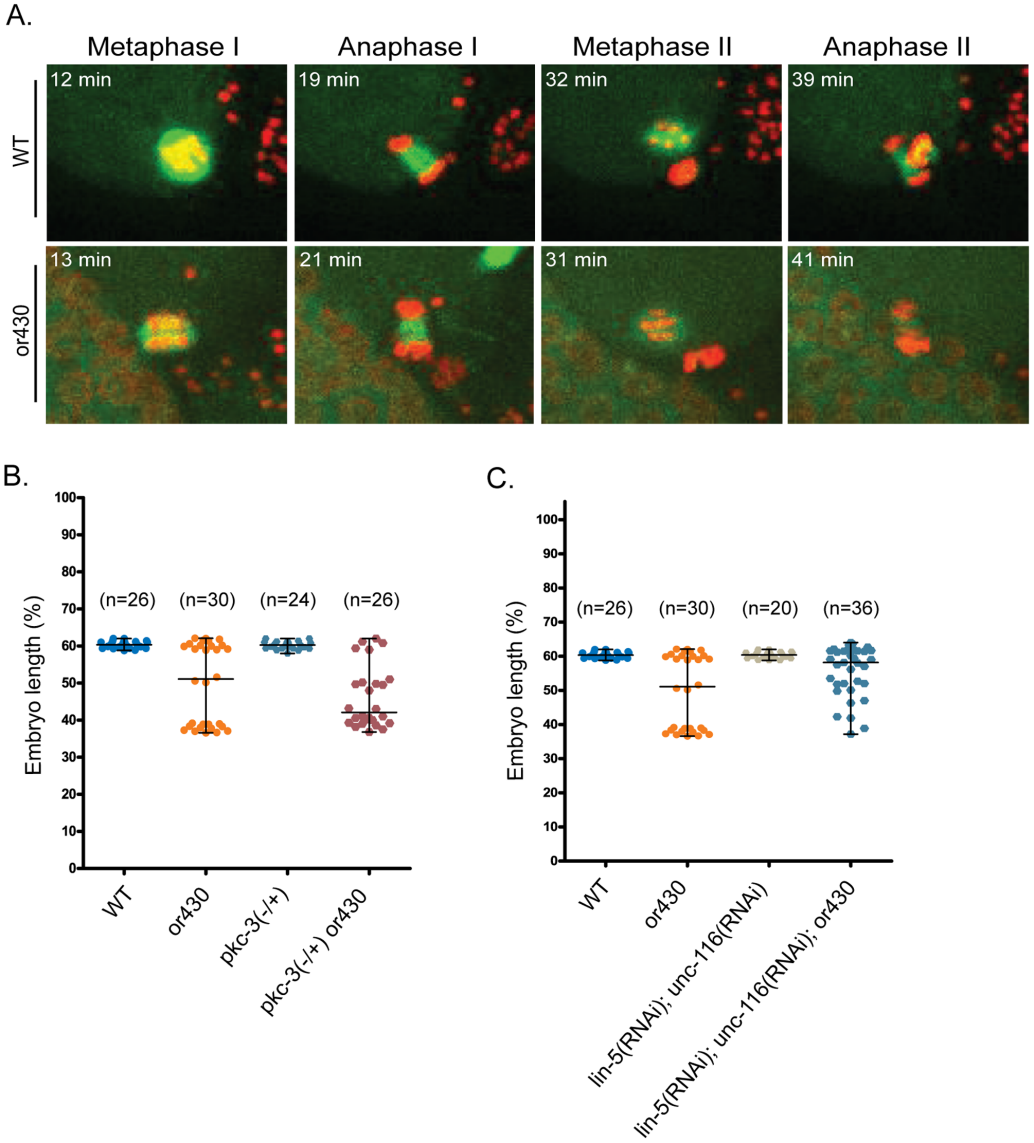
PKC-3 opposes and oocyte meiotic spindles promote AP axis reversal in *repo-1(or430*ts*)* zygotes. (A) Time-lapse confocal images of wild-type and *or430*ts oocyte Meiosis I and II in zygotes expressing GFP and mCherry fusions to ß-tubulin and Histone2B; t = 0 at ovulation. (B) P_0_ cleavage furrow position along AP after reducing *pkc-3* gene dosage. P = 0.006 for an independent t test to compare the difference of the furrow position for *or430* versus *or430; pkc-3(−/+)*. (C) P_0_ cleavage furrow position along AP axis after RNAi knockdown of *lin-5* and *unc-116*. P = 0.285 for an independent t test to compare the difference of the furrow position for *or430* versus *or430; unc-116(RNAi); lin-5(RNAi)*. WT and *or430*ts data in (B) and (C) are same as in Figure 1I.

Because the human ortholog of REPO-1, SF3a66, can bind to and bundle microtubules, we initially considered the possibility that *repo-1(or430*ts*)* might influence microtubule dynamics to cause a polarity reversal. Mutations that reduce the function of Anaphase Promoting Complex components arrest at metaphase of oocyte Meiosis I [19] and exhibit a partial reversal of AP polarity [15]. We therefore examined the timing of oocyte Meiosis I and II and found no differences in oocyte meiotic spindle morphology, or the timing of progression through Meiosis I and II, compared to wild-type (Figure 4A). While we cannot rule out more subtle defects in microtubule dynamics during oocyte meiotic spindle assembly and function, these results are suggest that the *repo-1(or430*ts*)* polarity reversals are not due to defects in microtubule dynamics during oocyte meiotic cell division.

### Reduced levels of PKC-3 may be responsible for AP polarity reversal in *repo-1(or430*ts*)* mutants

We next considered the possibility that the *repo-1(or430*ts*)* polarity reversals are caused indirectly by reduced levels of protein expression due to presumed splicing defects. Indeed, when examining GFP fusions to ß-tubulin, Histone 2B, and PAR-2, we consistently observed lower levels of fluorescence compared to wild-type embryos (Figures 1E, 1F, 2A, 2D). More specifically, we hypothesized that oocyte meiotic spindles in *repo-1(or430*ts*)* mutants might suffice to reverse polarity if levels of the anterior polarity protein PKC-3 were significantly reduced (see Introduction). We reasoned that if PKC-3 levels were abnormally low, the oocyte meiotic spindles might establish an anteriorly positioned cortical PAR-2 domain even in the absence of any delays through Meiosis I and II. Moreover, the mis-positioning of the sperm pronucleus-associated centrosomes and the apparent reduction in astral microtubules observed in some *repo-1(or430*ts*)* embryos (Figures 1C and 1F, data not shown) might compromise the ability of PAR-2 to load onto the cortex posteriorly, further contributing to the partially penetrant AP polarity reversal in *repo-1(or430*ts*)* mutants.

To test if reduced PKC-3 levels influence *repo-1(or430*ts*)* polarity reversals, we asked whether further reducing PKC-3 would increase the reversal penetrance. We constructed a strain homozygous for *repo-1(or430*ts*)* and heterozygous for the deletion allele *pkc-3(ok544)* and found that the penetrance of both polarity loss and reversal were significantly increased, from 10% (3/30) and 40% (12/30) to ∼23% (6/26) and ∼58% (15/26), respectively (Figure 4B). To test whether the polarity reversal also depends on microtubules associated with the oocyte meiotic spindle, we knocked down in *repo-1(or430*ts*)* mutants the genes *lin-5/NUMA* and *unc-116/*Kinesin 1, which bring the oocyte meiotic spindles into close proximity with the anterior cortex [20], [21]. We found that *lin-5/unc-116* knockdown led to a substantial decrease in the penetrance of polarity reversal, from 40% (12/30) to ∼17% (6/36), although this difference is not statistically significant (Figure 4C).

## Discussion

Based on our analysis of PKC-3 and oocyte meiotic spindles, it is possible that reduced levels of cortical PKC-3 activity or expression in *repo-1(or430*ts*)* mutants allow PAR-2 to better associate with the anterior cortex and reverse the AP axis of polarity, due to oocyte meiotic spindle microtubules protecting PAR-2 from PKC-3 phosphorylation, much as other microtubules normally appear to promote formation of a posteriorly positioned cortical PAR-2 domain. However, we have not directly examined the levels of PKC-3, and it also is possible that reduced levels of other proteins, such as the anterior polarity proteins PAR-3 or PAR-6, are more directly involved. Whether the polarity reversal is caused by reduced levels of PKC-3, or of other proteins, we think it is likely that the wild-type REPO-1/SF3a66 protein influences AP polarity only indirectly, through its function as a general splicing factor. We suggest that general splicing defects result in PKC-3 or other PAR polarity proteins being expressed at reduced levels in *repo-1(or430*ts) mutant embryos. These reduced protein levels then in turn may enable oocyte meiotic spindle microtubules to cause the polarity reversals observed in many *repo-1(or430*ts*)* mutant embryos. Consistent with a general splicing requirement for REPO-1/SFa66, *or430*ts mutants are defective in mitotic spindle assembly and progression through mitosis (our results) and in the innate immune response [22]. Thus we suspect that the AP axis reversal in *repo-1(or430*ts) mutants results indirectly from a general reduction in protein expression.

## Materials and Methods

### *C. elegans* strains and maintenance

Strains including wild-type N2 Bristol were grown under standard culture conditions [23]. *repo-1*(*or430*ts) was isolated in a screen for temperature-sensitive embryonic lethal mutations [16] and back-crossed six times with N2 males. Temperature-sensitive mutants were grown at 15°C and shifted to 26°C 2-3 hours before harvesting embryos for phenotypic analysis. Other strains and alleles used were: VC277 [pkc-3(ok544)/mIn1[dpy-10(e128) mIs14] II, DA491 [dpy-20(e1282) unc-30(e191)] IV, FX04961 (tm4961/+) IV, him-5(e1490) V. Transgenic GFP::β-tubulin and mCherry::Histone2B strains were derived from AZ244 (β-tubulin::GFP) and OD56 (mCherry::Histone2B). Transgenic GFP::PAR-2 and GFP::PGL-1 strains were derived from KK871 and JH227.

### Molecular Biology

*or430*ts was mapped to LG IV using visible markers *dpy-20* and *unc-30*. In 20/23 Unc-nonDpy recombinants, *or430*ts was linked to *dpy-20*; in 1/13 Dpy-nonUnc recombinants, *or430*ts was linked to *unc-30*. Sanger sequence analysis done by Sequetech.

*repo-1* RNAi was done by microinjecting dsRNA synthesized using T7 RiboMAX kits (Promega, Madison, WI) and diluted to 0.1 µg/µl [24]; phenotypes were analyzed 24 h post-injection. For other genes, feeding RNAi was used [25], [26]. For co-depletions, we transferred L4 larvae to plates seeded with an equal mixture of RNA-expressing *E. coli* strains.

### Live embryo imaging

For live imaging, embryos were mounted as described (16). Spinning disc confocal microscopy was done using a Leica DMI 4000B microscope fitted with a Leica 63X/1.40-0.60 HCX Plan Apo oil objective lens in a room maintained at 25°C. Images were obtained with a Hammamatsu EM-CCD digital camera using Volocity software (Perkin Elmer Inc.) and manipulated using ImageJ (http://rsb.info.nih.gov/ij/). To quantify PAR-2 levels in Figure 2, we used Image J to quantify GFP pixels for PAR-2: GFP in ∼25% of the entire cortex, where PAR-2 was mostly highly enriched, using identical focal planes within Z-stacks from both wild-type and or430 embryos. Meiosis I and II were imaged in live whole mount worms immobilized on 5% agar pads coated with 0.1 µm diameter polystyrene microspheres beads (Polysciences).
